# Role of Imaging in Screening for Hepatocellular Carcinoma

**DOI:** 10.3390/cancers16193400

**Published:** 2024-10-05

**Authors:** Irfan A. Kazi, Vinay Jahagirdar, Bareen W. Kabir, Almaan K. Syed, Asad W. Kabir, Abhilash Perisetti

**Affiliations:** 1 Department of Radiology, University of Missouri Columbia, Columbia, MO 65212, USA; f.kaziamirirfan@health.missouri.edu; 2 Division of Gastroenterology, Hepatology and Nutrition, Virginia Commonwealth University, Richmond, VA 23298, USA; vinay.jahagirdar@vcuhealth.org; 3 Department of Internal Medicine, University of Missouri Columbia, Columbia, MO 65212, USA; b.kabir@health.missouri.edu; 4 Blue Valley Southwest High School, Overland Park, KS 6622, USA; aksyed2@gmail.com; 5 Mosaic Life Care, St. Joseph, MO 64506, USA; asad.kabir@mymlc.com; 6 Division of Gastroenterology and Hepatology, Kansas City Veteran Affairs, Kansas City, MO 64128, USA

**Keywords:** hepatocellular carcinoma, chronic liver disease, HCC screening ultrasound, HCC screening MRI, Biomarkers in HCC screening, HCC Diagnosis

## Abstract

**Simple Summary:**

Liver cancer ranks as the sixth most common type of cancer and is the third leading cause of cancer deaths. The most frequent type of liver cancer is hepatocellular carcinoma (HCC), making up about 90% of these cases. HCC mainly occurs in people who have cirrhosis, a serious liver condition often caused by chronic liver diseases like hepatitis B and C or long-term heavy alcohol intake. Currently, due to the rise in obesity, more people are being diagnosed with nonalcoholic fatty liver disease, which can also lead to cirrhosis. Since liver cancer often develops in those with chronic liver issues, early screening in at-risk groups is crucial for better outcomes. It is important to note that about 20% of HCC cases occur in people without cirrhosis, which can sometimes lead to delayed screening and worse outcomes. This article also covers the screening methods for liver cancer in people with cirrhosis.

**Abstract:**

Primary liver cancer is among the most common cancers globally. It is the sixth-most common malignancy encountered and the third-most common cause of cancer-related death. Hepatocellular carcinoma (HCC) is the most common primary liver malignancy, accounting for about 90% of primary liver cancers. The majority of HCCs occur in patients with underlying cirrhosis, which results from chronic liver diseases such as fatty liver, hepatitis B and hepatitis C infections, and chronic alcohol use, which are the leading causes. The obesity pandemic has led to an increased prevalence of nonalcoholic fatty liver disease (NAFLD), which leads to nonalcoholic steatohepatitis and could progress to cirrhosis. As HCC is among the most common cancers and occurs in the setting of chronic liver disease in most patients, screening the population at risk could help in early diagnosis and management, leading to improved survival. Screening for HCC is performed using biochemical marker testing such as α-fetoprotein (AFP) and cross-sectional imaging. It is critical to emphasize that HCC could potentially occur in patients without cirrhosis (non-cirrhotic HCC), which can account for almost 20% of all HCCs. The lack of cirrhosis can cause a delay in surveillance, which could potentially lead to diagnosis at a later stage, worsening the prognosis for such patients. In this article, we discuss the diagnosis of cirrhosis in at-risk populations with details on the different modalities available for screening HCC in patients with cirrhosis, emphasizing the role of abdominal ultrasounds, the primary imaging modality in HCC screening.

## 1. Introduction

Primary liver cancer is among the leading causes of malignancy and cancer-related mortality, the sixth most diagnosed cancer and third most common cause of cancer-related mortality [1]. Hepatocellular carcinoma (HCC) is the most common primary liver cancer, constituting approximately 90% of liver-related malignancies [1,2,3]. Although HCC can arise without an underlying cause, about 80–90% are associated with chronic liver disease secondary to a known risk factor. Cirrhosis, which arises from chronic liver disease, is the most common cause of HCC. Typically, any cirrhosis-inducing condition could increase the risk for the development of HCC. Hepatitis B, hepatitis C, alcoholic liver disease, and nonalcoholic fatty liver disease are the leading causes of chronic liver disease and the development of HCC [1,2,4,5].

Multiple factors determine the development of chronic liver disease and HCC, including the geographic prevalence of risk factors, social and environmental determinants, and genetic susceptibility [4]. Specifically, the highest incidence of HCC is seen in East and Southeast Asia and North and West Africa, secondary to the high prevalence of hepatitis B and C infections in these regions, which contribute to 80% of HCC cases worldwide [6,7]. In the United States (US), the Asian American population had the highest incidence of HCC, followed by African American, Hispanic, and non-Hispanic white populations, respectively. The Asian American population had a two-fold risk for the development of HCC compared to African Americans, and the African American population has twice the risk when compared to the non-Hispanic white population, respectively. In a person who has the risk factors or has developed chronic liver disease, socioeconomic status, access to healthcare, and genetic susceptibility play a crucial role in the prevention of HCC, early diagnosis, and appropriate management [8].

The incidence of liver cancer, cancer-related deaths, and the crude number of disease-adjusted life years (DALYs) significantly increased in 1990–2019. There were estimated DALYs of 11,278,630 in 1990 and 12,528,422 in 2019, and liver cancer-related mortality of 365,215 in 1990 and 484,577 in 2019 [7]. Data from a global database suggest that an estimated 905,700 individuals were diagnosed with liver cancer worldwide, and 830,200 deaths were attributed to the disease in 2020 [1]. Primary liver cancer stands not only as one of the most common causes of cancer-related mortality but also ranks as the second leading cause of premature death globally [9]. Due to increasing awareness about the spread of hepatotropic viruses (hepatitis B and C), the implementation of robust vaccination programs, and the development of direct-acting antiviral (DAA) medications, there has been an overall decline in the trend in HCC secondary to these viral infections [10,11,12]. However, this trend has been offset by the increasing cases of HCC caused by nonalcoholic fatty liver disease (NAFLD) [3,13,14]. In the Western population, chronic HCV infection is the most common cause of HCC, with about 20% of the cases caused by HBV infection [15].

## 2. The Rationale for Screening of HCC

A screening program aims to detect disease at a stage when early intervention helps achieve a better outcome [16]. The disease in question should constitute a significant public health problem and have available treatment, with increased potential for cure with early detection [17]. HCC is among the commonly encountered cancers and the leading causes of cancer-related mortality, thus constituting a significant public health problem. Patients with an early diagnosis of HCC have a better prognosis (better curative treatment options) with five-year survival rates exceeding 70% [18]. However, in patients with advanced HCC, the prognosis is significantly reduced to less than 20% [19]. Hence, early detection via better screening strategies could help detect HCC promptly, thereby improving patient outcomes.

## 3. Diagnosis of Chronic Liver Disease and Cirrhosis

An early diagnosis of chronic liver disease and cirrhosis helps in early intervention and appropriate management. It is essential to identify the possible etiology of chronic liver disease. A thorough evaluation for chronic liver disease is needed, including screening tests for HBV, HCV, serological workup, and alcohol and drug use. When the precise cause of chronic liver disease is not apparent, further evaluation (such as liver biopsy) may be needed. Evaluation for autoimmune diseases and other rare hepatic diseases may be required, including α1-antitrypsin deficiency, hemochromatosis, and Wilson’s disease. A liver biopsy may be needed when a noninvasive workup does not determine the cause (and if more than one diagnosis is possible and treatment of one etiology could worsen the other etiology) of chronic liver disease [20,21].

In addition to identifying the cause of chronic liver disease, its severity also must be assessed. Chronic liver disease can remain asymptomatic until decompensation in the form of ascites, hepatic encephalopathy, or variceal bleeding or can be complicated by the development of HCC. Only about 33% of the population with cirrhosis are aware of their diagnosis [22]. Chronic liver disease is evaluated by liver function tests, complete blood count with platelets, prothrombin time, and abdominal sonography. Further investigations can be performed based on the patient’s risk factors and demographics [20,23,24]. HCC is predominantly linked with cirrhosis, with about 80% of HCCs arising in patients with cirrhotic livers. However, approximately 20% of HCC cases occur without cirrhosis, classified as non-cirrhotic HCC (NCHCC) [25]. The absence of cirrhosis in NCHCC can lead to a lack of regular surveillance, often resulting in a later-stage diagnosis and, consequently, a poorer prognosis compared to HCC cases with underlying cirrhosis (CHCC) [26].

Hepatic parenchymal fibrosis is the hallmark of most chronic liver diseases and one of the most important prognostic factors of chronic liver disease, including its related complications. Standard testing to assess the extent of hepatic fibrosis and the development of cirrhosis includes serum biomarkers and transient elastography [22,24]. Hepatic fibrosis is scored from F0 to F4, with a score of F0 implying no hepatic parenchymal fibrosis and a score of F4 suggesting advanced liver fibrosis. A score of F2 or higher implies significant hepatic fibrosis. Transient elastography is not accurate in the presence of ascites and obesity (due to the inability to identify the liver accurately) [22,24]. When transient elastography is not adequate, liver fibrosis may be quantified by ultrasound shear wave elastography or magnetic resonance (MR) elastography. MR elastography is the most accurate noninvasive imaging modality for evaluating hepatic fibrosis, but it is available only in specialized centers [24,27,28]. Generally, the noninvasive tests, including the serum biomarkers and imaging are most accurate in identifying patients with no to minimal fibrosis and in patients with advanced fibrosis. They are less precise in determining the intermediate stages of liver disease [22]. A liver biopsy is considered the gold standard for evaluating hepatic fibrosis. However, it is an invasive procedure with a risk of rare but potentially life-threatening complications [29]. Noninvasive tests are the mainstay in diagnosing chronic liver disease, with liver biopsy as a problem-solving tool [20,21,24]. While noninvasive methods have increasingly substituted for or complemented a significant portion of liver biopsies, some societies still recommend considering liver biopsy in patients who exhibit typical imaging findings but lack risk factors, as well as in those who have risk factors but do not display typical imaging findings [30]. This approach ensures that diagnostic accuracy is maintained, particularly in atypical presentations. In a prospective, observational study, liver biopsy was conducted on 81 patients with chronically abnormal liver function of unknown origin. The biopsy results revealed simple steatosis in 41 patients and NASH in 26 patients, underscoring the significance of performing a liver biopsy in the diagnostic process [31].

## 4. At-Risk Population at Risk for Development of HCC

Hepatitis B viral infection (HBV): Hepatitis B viral infection is the leading cause of the development of HCC. In 2018, about 360,000 cases of HCC were secondary to HBV infection, constituting 55% of the total cases of HCC [32].

Hepatitis B is classified as a class I carcinogen, and chronic infection with it carries a lifetime risk of 10–25% for developing HCC. The development of HCC can occur directly through integrating the hepatitis B virus’s covalently closed circular DNA (cccDNA) into the genome of the host’s hepatocytes, which may trigger the activation of oncogenes. Indirectly, the risk of HCC is heightened due to the immune response activation leading to inflammatory damage to liver cells, fibrogenesis, and the subsequent regeneration of hepatocytes [33]. The risk of HCC is closely linked to persistent hepatitis B infection, and there is typically a period of thirty to forty years between the onset of infection and the development of HCC [33,34,35].

About 10% of the population infected with HBV develop chronic infection. Chronic HBV infection results in chronic liver disease due to cycles of necrosis, inflammation, and regeneration, culminating in cirrhosis, thus increasing the risk for HCC [4]. In established cirrhosis secondary to HBV, the annual incidence of HCC is 2–5% [32]. HBV DNA can integrate into the host DNA and produce a protein HBx, which can activate protooncogenes and inhibit the activity of tumor suppressor genes, leading to the development of HCC even in the absence of cirrhosis. This increased risk shows a dose–response relationship with HBV viral DNA level, with increased viral DNA level increasing the risk of developing HCC [4,5,32,36]. In chronic HBV infection, transient elastography is more accurate than serum biomarkers in diagnosing advanced fibrosis and cirrhosis [24].

Numerous critical elements, such as the age at which HBV transmission occurs, environmental influences, viral genotype, family history, and other factors, contribute to the varied interactions between the immune system, genomic alterations, and the development of HCC on an individual basis. These factors should be considered to accurately conduct HCC surveillance tailored to the individual risk profiles within a population [37].

Hepatitis C viral infection (HCV): HCV is an RNA virus that can quickly mutate and evade the host’s immune system. About 60 to 80% of people infected with HCV develop chronic infection in comparison to 10% for HBV infection. In 2018, about 160,000 cases of HCC were secondary to HCV infection, constituting 25% of the total cases of HCC [32]. The population infected with HCV is also more prone to the development of cirrhosis when compared to HBV infection. After ten years of infection, about 5–10% of the population infected with HCV develop cirrhosis, which is 10–20-fold higher when compared to HBV infection [4]. Oxidative stress due to chronic HCV infection leads to the development of chronic liver disease, resulting in cirrhosis with an increased risk of developing HCC [4,5,32]. Once cirrhosis develops in the setting of HCV infection, the annual incidence of developing HCC is 2–4% [32]. In evaluating chronic liver disease secondary to chronic HCV infection, transient elastography, and serum biomarkers are equally accurate in detecting significant fibrosis/cirrhosis [24].

The advent of safe and efficacious directly acting antiviral (DAA) medications has made it possible to eradicate the infection in nearly all patients who undergo treatment. It has led to a substantial reduction, although not a complete elimination, in the risk of hepatocellular carcinoma (HCC) in those who have been cured of the virus [38]. A systematic review indicated that in patients with cirrhosis, the incidence of HCC declines over time following the cure of HCV infection [39].

Alcohol-related liver disease: Chronic alcohol consumption accounts for about 17% of cases of liver cancer globally [1]. Alcohol-related cirrhosis is the most important cause of HCC in areas with a low prevalence of HBV and HCV infections [40]. The incidence of HCC due to alcoholic liver disease has demonstrated an increasing trend from 1990 to 2019 [7]. Alcohol is predominantly metabolized in the liver, and the severity of alcohol-related liver disease is directly proportional to the dose of alcohol consumed. Alcoholic liver disease (ALD) causes hepatic injury, which ranges in severity from biochemical damage, alcoholic fatty liver disease, alcoholic hepatitis, hepatic parenchymal fibrosis, and finally leading to cirrhosis. Women are more susceptible to the development of ALD for the same amount of alcohol consumption and can develop the disease earlier and with smaller quantities of alcohol when compared to the male population. Genetic susceptibility also plays a role, and some genetic polymorphisms have been identified, which increase the susceptibility for developing chronic liver disease with alcohol abuse. Chronic alcohol abuse causes oxidative stress with cycles of hepatic inflammation, necrosis, and regeneration, resulting in chronic liver disease and, ultimately, cirrhosis [4,40]. In patients with ALD, transient elastography is accurate in ruling out severe fibrosis or cirrhosis [24].

Nonalcoholic fatty liver disease: Nonalcoholic fatty liver disease (NAFLD) is defined as a condition in which more than 5% of hepatocytes demonstrate macrovesicular steatosis in the setting of a patient population where an alternative cause of fat deposition is not readily identified and who drink little or no alcohol. Like ALD, NAFLD can cause a spectrum of diseases ranging from hepatic steatosis, nonalcoholic steatohepatitis (NASH), hepatic parenchymal fibrosis, and cirrhosis [7,41]. The increasing prevalence of obesity and metabolic syndrome worldwide has led to a rise in NAFLD. NAFLD is estimated to affect 25–30% of the general population [41]. About 10–30% of the cases of NAFLD progress to cirrhosis [15]. It is expected that NASH-related cirrhosis will be the leading cause of cirrhosis in patients awaiting liver transplantation in the United States sometime between 2025 and 2035 [22]. It is estimated that about six million people have NASH in the United States. Although people with NASH-related cirrhosis have a much higher likelihood of developing HCC when compared to the patient population having non-cirrhotic NASH, more than 25% of NASH-related HCC occurs before cirrhosis has developed due to the large patient population affected by NASH. However, the annual incidence rate of HCC in non-cirrhotic NASH is low [15]. A meta-analysis involving 470,404 patients reported that patients with cirrhosis related to NAFLD face a risk of developing HCC that is comparable to the risk reported for patients with cirrhosis from other causes [42]. Patients with NAFLD should be screened for liver fibrosis, especially those with metabolic syndrome or type 2 diabetes mellitus, as they have an increased risk for hepatic fibrosis. The screening for hepatic fibrosis can be performed using serum biomarkers or transient elastography in patients at low risk. Patients with NAFLD should be screened at a three-year interval for progression of hepatic fibrosis using either serum biomarkers or transient elastography [24].

In addition to the above-mentioned common causes of chronic liver disease, cirrhosis from other causes also increases the risk of developing HCC, and surveillance is needed for early diagnosis and prompt management [22,24,43]. Once the diagnosis of cirrhosis has been made, the severity can be scored based on criteria. The most common scoring systems used in classifying the severity of cirrhosis include the Child–Pugh score and the Model for End-stage Liver Disease. Based on the Child–Pugh Score, cirrhosis can be grouped into Class A, B, and C. Child–Pugh Class C has a poor prognosis with a 2-year survival of 35% [44]. The risk factors for NCHCC commonly include nonalcoholic fatty liver disease (NAFLD) and viral hepatitis infections (hepatitis B virus (HBV) and hepatitis C virus (HCV)) [36]. The imaging characteristics of CHCC and NCHCC share many similarities, but some differences exist. CHCC typically develops in a cirrhotic liver, which can be identified on imaging, whereas NCHCC does not have this background of cirrhosis. While CHCC can present with multiple masses, NCHCC usually has a large solitary or dominant mass with satellite nodules [45]. NCHCC is more likely to exhibit extrahepatic extension with direct invasion into adjacent organs [5]. Encasement or invasion of the blood vessels is more commonly associated with CHCC [5].

## 5. Screening for HCC Using Imaging

The target population to be screened for HCC includes Child–Pugh Class A and B cirrhosis of any etiology, Child–Pugh Class C cirrhosis patients who are transplant candidates, and a specific non-cirrhotic chronic HBV-infected population. As discussed earlier in the present article, patients with chronic HBV infection are prone to develop HCC even without cirrhosis. The population of non-cirrhotic HBV infection who should be screened for HCC include Asian men more than 40 years of age, Asian women more than 50 years of age, and those with a family history of HCC. People from Africa and the North American black population need to be surveilled earlier as they develop HCC at a much younger age [43].

An ideal screening test should accurately detect a disease in its preclinical phase. It should detect the disease during the phase where treatment is more effective. The test should be safe for the patient, inexpensive, and widely available [17]. Screening can be performed using biochemical markers or imaging as stand-alone modalities or combined. The most accepted screening tool is abdominal ultrasound. Biochemical screening can be performed using serum α-fetoprotein (AFP levels). Multiple biomarkers are being evaluated for HCC surveillance. However, these are still in the trial phase. In addition to abdominal ultrasound, imaging surveillance can also be performed on Computed Tomography (CT) or Magnetic Resonance Imaging (MRI) of the abdomen [43].

## 6. Abdominal Ultrasound

Abdominal ultrasounds are the most used imaging tool in the screening for HCC. Ultrasounds are a noninvasive, nonionizing, cost-effective imaging modality with widespread availability [46]. The role of ultrasounds in screening for HCC has been validated in multiple studies. It was found to be 94% sensitive for the detection of HCC. However, in the case of small-sized lesions, the sensitivity of ultrasounds fell to 63% [47]. Another study reports 84% and 47% sensitivity for detecting HCC and early HCC, respectively [47,48]. The role of ultrasounds in screening HCC is to detect lesions and not to characterize them [49].

Ultrasounds are an operator-dependent modality. Ultrasound imaging needs to be optimized for a thorough evaluation of the liver. It is preferably performed after 4–6 h of fasting to avoid a suboptimal liver window by the overlying bowel gas. A curvilinear transducer is typically used, and a high-frequency linear transducer may be used to evaluate superficial hepatic parenchyma if required. The examination is initially performed while the patient is lying supine. Additional views in the left lateral decubitus and left posterior oblique positions may be required for complete visualization of the liver parenchyma. The evaluation should be performed using both intercostal and subcostal approaches. The superior aspect of the liver is best evaluated in deep inspiration. Care should be taken to adequately assess the left lobe of the liver, which lies in the epigastric region. A systematic approach and evaluation of both longitudinal and transverse views are essential to avoid missing lesions. The portal and the hepatic venous systems should be evaluated for patency. Ultrasounds are also helpful to corroborate the findings of cirrhosis and can demonstrate signs of portal hypertension such as ascites, splenomegaly, and portosystemic venous collaterals. In chronic liver disease, a thorough evaluation of liver parenchyma can be limited by multiple factors. A few factors are intrinsic to the liver in chronic liver disease. These include coarse hepatic echotexture, hepatic parenchymal nodularity, and the fibrotic liver’s attenuation of the sound waves. Patients may have co-existing hepatic steatosis, especially seen in patients with cirrhosis secondary to ALD and NAFLD, which, depending upon the severity, also decreases the sensitivity of the examination. A shrunken liver may be present, which also impairs visualization. Factors extrinsic to the liver impairing visualization include large body habitus, overlying bowel gas, and patients’ inability to follow instructions [19,28,49]. 

**Figure 1:**
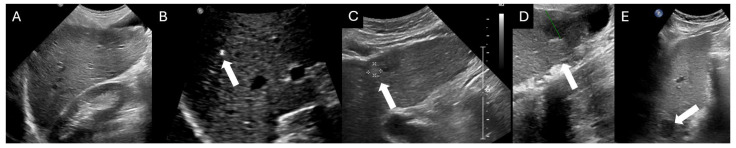
Ultrasound of the liver in five patients with chronic liver disease with respective US LI-RADS scores. Image (**A**) demonstrates no focal abnormality and corresponds to a US LI-RADS score of 1. Follow-up US needs to be performed every six months. Images (**B**,**C**) demonstrate subcentimeter observations measuring 0.29 cm and 0.8 cm respectively (arrows), which are assigned a US LI-RADS score of 2. Short-term follow-up examinations need to be performed to look at the evolution of these observations. Images (**D**,**E**) demonstrate observations larger than 1 cm (arrows), which are assigned a US LI-RADS score of 3. These need to be characterized further on multiphasic CT or MRI.

**Figure 2:**
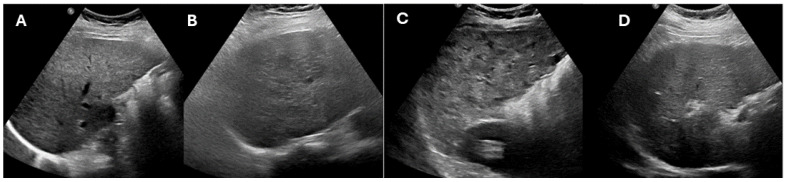
Ultrasound of the liver in four patients with chronic liver disease with the Ultrasound LI-RADS visualization score examples. Image (**A**) demonstrates coarse hepatic echotexture. The entire hepatic parenchyma is optimally visualized, which corresponds to a US visualization score of A. In image (**B**), the hepatic parenchyma appears heterogeneous, and evaluation for small hepatic observations is suboptimal. This corresponds to the US visualization score B. In image (**C**), the hepatic parenchyma is markedly coarsened, severely limiting evaluation for focal hepatic lesions. This corresponds to the US visualization score C. In Image (**D**), the hepatic parenchyma close to the diaphragm (along the inferior aspect of the image) is suboptimal visualized, which severely limits the evaluation. This corresponds to a US visualization score of C.

The American College of Radiology (ACR) has developed a standardized reporting algorithm for screening HCC in the setting of cirrhosis, known as the Liver Imaging Reporting and Data System (LI-RADS) (Figure 1 and Figure 2). The initial algorithm was developed for CT and MRI lesion characterization. The Ultrasound Liver Imaging Reporting and Data System (US LI-RADS) was created in 2017 [50]. The US LI-RADS has two components: A. Detection of focal abnormality and B. Assessment of the visibility of hepatic parenchyma. A. Detection of focal abnormality: When a focal area differs from the adjacent parenchyma, it is labeled as an observation because it can be either a non-neoplastic or a neoplastic process. The terms lesion or mass, which signify a pathological process, are avoided. Examples of observations include solid nodules or focal areas of ill-defined borders/architectural abnormality.

Based on the ultrasound findings, three US LI-RADS categories are possible. Category US-1: Negative, implying no evidence of HCC; other benign observations may or may not be present. If there is a solid observation, it should have been proven benign on prior post-contrast CT or MRI. Category US-2: Subthreshold, implying that there is an observation that measures less than 10 mm. Observations less than 10 mm are often seen on ultrasounds and are generally benign. These are also difficult to evaluate in CT or MRI studies. They should be followed up on serial studies for up to 2 years before being labeled benign. If there is an interval increase in size, they are labeled as US-3 observations. Category US-3: Positive, includes focal solid observation greater than 10 mm, which was not characterized as benign, or geographic areas of architectural distortion greater than 10 mm. This category also includes new thrombus in the portal or hepatic venous systems. Whenever there is a doubt as to which category to be assigned, the higher of the two categories should be selected. B. Assessment of the visibility of hepatic parenchyma: As discussed earlier, evaluating the entire liver for focal lesions may be suboptimal, secondary to multiple factors. A “Visualization Score” is assigned to objectively assess the examination’s completeness. Score A implies that there were no or minimal limitations to the evaluation. Score B means that limitations are present in the study, and there is a chance that small observations may not be picked up on the examination. Score C implies that there are limitations that significantly reduce the examination’s sensitivity for focal liver observations. The US visualization score is a guide for the diagnostic certainty of the examination; however, no recommendations are made if there is a severely limited examination, and the US LI-RADS category dictates further evaluation. Based on US LI-RADS categorization, if there is a US-1 examination, routine surveillance is recommended every six months. If there is a US-2 observation, a short-term repeat ultrasound examination should be performed between 3 and 6 months. A US-3 observation should be characterized further on a multi-phase contrast-enhanced CT or MRI study [15,19,28,43,49,50].

## 7. CT and MRI

HCC is distinct from other tumors because its diagnosis can often be established based solely on radiologic criteria, utilizing dynamic contrast-enhanced CT or MRI [51,52]. While biopsy can be employed for cases presenting atypical imaging features, histological confirmation is unnecessary for most cases [53]. Compared to ultrasounds, multiphasic CT and MRI studies have superior sensitivity for detecting early HCCs. CT involves radiation, and nonenhanced CT is suboptimal in identifying focal liver lesions. A multiphasic CT study, which involves intravenous contrast injection, needs to be performed for optimal screening. The multiphasic CT study increases the radiation dose. Also, CT is a costlier tool than ultrasounds. It is therefore not advisable to use CT as a primary screening modality every six months [15,19,54]. 

MRI avoids radiation and is superior for lesion characterization. MRI as a screening tool has the disadvantages of an extended examination duration and increased cost of screening. MRI is an exceptional imaging modality for detecting and characterizing lesions, thanks to its superior contrast resolution and ability to evaluate a broader range of tissue properties beyond just vascularization [55]. A meta-analysis reported that for the diagnosis of hepatocellular carcinoma, the pooled sensitivity and specificity were 70% and 94%, respectively, which was independent of the tumor size [56]. The sensitivity was greater for lesions larger than 2 cm (close to 100%), but it reduced to 58.3–64.6% for lesions that were smaller than 2 cm. For lesions smaller than one centimeter, the sensitivity was even lower [57,58,59]. The size of an HCC lesion is an independent prognostic factor and is relevant in all staging systems. Contrast-enhanced MRI using hepatospecific contrast agents, specifically gadoxetate disodium and gadobenate dimeglumine, is indispensable in the diagnosis of HCC as it can further enhance sensitivity by an additional 5–10% [58,60,61].

Abbreviated MRI protocols have been suggested, which decrease the examination’s duration. In the abbreviated MRI protocol, only sequences required for HCC detection are used. Three approaches have been proposed to shorten the duration of MRI. The first protocol performs a non-contrast MRI using T1, T2, and diffusion-weighted sequences. The second approach includes acquiring T1-weighted pre-contrast and dynamic post-contrast images in the arterial phase, portal venous phase, and delayed phase. In the third abbreviated MRI screening technique, imaging is performed using hepatobiliary contrast. Images are acquired 20 min post-injection of the hepatobiliary contrast. T1 and T2, and diffusion-weighted sequences are acquired [28,62]. If a focal lesion is seen, it would still need to be characterized in detail on a complete MRI examination [19,28,49]. Therefore, although CT and MRI studies are more accurate, they are not primarily used in HCC screening.

## 8. Screening Using Biomarkers

A wide array of biomarkers have been studied for use in the screening of HCC. These can be classified into embryonic antigens, protein antigens, enzymes and isoenzymes, growth factors on the receptors, cytokines, metabolites, and molecular markers (for example, cell-free DNA, mRNA, miRNA, etc.). The biomarkers can be used either as a stand-alone, in combination with other biomarkers, or other diagnostic modalities [63].

The most studied biomarker in HCC screening is AFP. It is inexpensive, readily available, and simple to perform. It is limited by low sensitivity and specificity when used as a stand-alone tool for HCC screening [18,43,49]. More than 40% of HCCs have normal AFP levels, and AFP values can also be raised in other cancers [43]. Several other markers have been proposed and are under investigation, either alone or in combination, but they are still under trial and are not available for routine screening. They include cell-free DNA, AFP-L3, an AFP subfraction, and des gamma carboxy prothrombin (DCP) [43]. A GALAD score, which combines the patient’s gender, age, AFP level, AFP-L3%, and DCP, is associated with a 5-fold increased risk for the development of HCC. Many other biomarkers have been used either alone or in combination. However, none of the newer biomarkers have been validated in large-scale prospective studies [64].

Although AFP cannot be used as a stand-alone tool for detecting HCC, adding AFP to ultrasounds in HCC screening has been superior to using ultrasounds alone [19,43]. Ultrasounds alone possess a low sensitivity for detecting early-stage HCC in patients with cirrhosis [48]. The findings of a meta-analysis suggested that including AFP testing alongside ultrasounds significantly enhances the sensitivity for early detection of HCC in clinical settings [48]. Considering both the benefits and harms related to surveillance, utilizing ultrasounds in conjunction with AFP testing proved to be a more cost-effective approach for monitoring HCC than relying on ultrasounds alone or foregoing surveillance entirely in individuals with compensated cirrhosis [65].

## 9. Cost-Effectiveness of HCC Surveillance

Parikh et al. [65] in their cost effectiveness analysis of HCC surveillance, compared ultrasounds, ultrasounds plus AFP, and no surveillance. They concluded that a combination of ultrasound and AFP was more cost-effective when compared to ultrasound alone, or to no surveillance being conducted. They noted that an incidence of HCC greater than 0.4%/year and adherence to surveillance, which would be greater than 19.5% biannually, was required for the combination of ultrasound and AFP to be cost-effective when compared to no surveillance being carried out [65]. Many studies have demonstrated that HCC screening is cost-effective. However, Nguyen et al. [66] reported that there are significant limitations in these studies. Therefore, the results of these studies should be interpreted with caution. Robust studies should be performed in the future that take into account the key factors in relation to HCC surveillance, including true utilization rates of the surveillance modalities and the predicted increasing incidence of HCC over time [66].

## 10. Screening Anxiety

Informing patients with chronic liver disease about the risk for the development of HCC can lead to the development of anxiety, emotional distress, and apprehensions. Patient concerns need to be identified and promptly addressed. Patient counseling is necessary to educate them about the benefits of screening and to avoid the psychological harm that may occur as a result of the screening [67,68]. Several studies have shown that monitoring for HCC enhances early tumor detection and overall survival rate [69,70]. Studies on other types of cancer indicate that positive screening outcomes can lead to psychological distress, such as depression, anxiety, and reduced quality of life, both immediately and over time [71]. The emotional impact of HCC monitoring might vary since it is conducted in populations with a higher risk of cancer. Additionally, patients with cirrhosis often suffer from mental health issues, with about one-sixth experiencing moderately severe depression and almost half suffering from moderate to severe anxiety [72]. A recently published multicenter randomized trial focusing on HCC surveillance outreach indicated that while the psychological impact of HCC screening is generally mild, it varies depending on the screening outcome [67]. Patients with true negative test results generally showed a decrease in depression and anxiety over time. In contrast, those with true positive test results experienced an increase in depression following the diagnosis of HCC. Thus, the psychological consequences should be considered when evaluating the benefits of HCC surveillance programs.

A prospective survey involving patients at risk for HCC from multiple centers indicated a strong preference for early detection of HCC, outweighing concerns over potential surveillance-related harms or inconveniences. Patients preferred abbreviated MRI, complete MRI, or new blood-based biomarkers over ultrasounds alone or ultrasounds combined with AFP testing [73]. This demonstrates a significant interest in more advanced and potentially more effective methods for early HCC detection among at-risk populations.

## 11. Screening Recommendations and Improving HCC Screening

Screening for HCC has been recommended by multiple societies, such as the American Association for the Study of Liver Diseases (AASLD), the European Association for the Study of the Liver, the Japan Society of Hepatology (JSH), and the Asian Pacific Association for the Study for the Liver (APASL). The AASLD recommends that HCC surveillance be performed every six months and that ultrasounds and AFP be combined for screening [24]. As per the European Association for the Study of Liver (EASL), ultrasound evaluation is recommended every six months [74]. When there is poor visualization of the hepatic parenchyma on ultrasounds (LI-RADS Visualization score C), the American Association for the Study of Liver Diseases (AASLD) has recommended that multiphasic contrast-enhanced CT/contrast-enhanced MRI be considered for some patients as needed [43]. All societies recommend using ultrasounds as the first imaging modality at intervals of 4–8 months (with a mean of every 6 months).

Typically, of the high-risk population who would benefit from screening for HCC, less than 5% of the patient population who qualify for screening undergo the biannual surveillance as per the recommended guidelines [75]. In a recently published cohort study focusing on patients with cirrhosis, it was found that approximately two-thirds of the patients with HCC did not undergo prior surveillance, highlighting significant shortcomings throughout the screening process. There is a clear need to understand the underlying barriers and implement dedicated programs to enhance HCC surveillance [76].

Primary care providers (PCPs) can have misconceptions and apprehensions regarding the tests that can detect hepatocellular carcinoma (HCC), which can lead to ineffective surveillance strategies. A survey covered 131 primary care providers in the setting of a large urban hospital in the United States in 2015. It reported that 65% of the providers conducted annual surveillance, and only 15% carried out biannual surveillance [77]. In another survey of 391 PCPs from North Carolina, although 89% were taking care of patients with cirrhosis, less than half admitted to screening for HCC [78]. To enhance early detection of HCC, the National Cancer Institute launched the Translational Liver Cancer (TLC) consortium in 2018. The consortium aims to refine the risk stratification of patients for developing HCC, enhance screening processes within high-risk groups, and boost the rate of early-stage HCC detection by evaluating innovative screening strategies like abbreviated MRI sequences. It also works to establish large patient cohorts to validate biomarkers and imaging techniques [79].

There is significant interest in employing artificial intelligence (AI) technology for cancer screening and detection. For instance, in recent years, the US Food and Drug Administration (FDA) has approved several advanced AI products specifically for use in breast cancer screening (BCS) [80,81]. With the advent of artificial intelligence (AI) and a vast amount of imaging data, there has been rapid advancement in the development of machine learning and deep learning algorithms to diagnose chronic liver disease and hepatocellular carcinoma on ultrasounds, CT, and MRI. Omics is the usage of data analysis to study biological systems. Radiomics involves extracting quantitative data from imaging. The data obtained on imaging is analyzed using machine learning and feature extraction algorithms to generate quantitative information that is not apparent to the human observer. Multiomics involves integrating computational data obtained from different omics and leveraging the information to optimize the clinical question that needs to be addressed [82]. Multiomics can improve HCC screening by integrating radiomics with genomics, proteomics, and metabolomics. Algorithms have also been developed for treatment planning, estimating the process, and predicting tumor recurrence [83].

Zhang et al. used a deep learning model to identify focal hepatic lesions on ultrasounds in patients with chronic hepatitis B infection. They achieved an accuracy of 94% in the test cohort [84]. Yu et al. used a panel of fusion genes found in HCC at varying degrees of frequency in combination with serum AFP levels, which were incorporated into a machine-learning model. This model demonstrated an accuracy of 95% for HCC detection [85]. Liang et al. used a deep learning algorithm based on electronic health records, imaging, histopathology, and molecular biomarkers [86]. Their algorithm showed an AUC of 0.94 for predicting a 1-year risk of HCC. Singal et al. used a machine learning algorithm utilizing patient demographics, clinical data, and laboratory parameters in patients with Child A or B cirrhosis. Their algorithm had a c-statistics score of 0.64 [87]. Utilizing computer-aided diagnosis (CAD) systems on multi-phase computed tomography (CT) scans for detecting hepatocellular carcinoma (HCC), Lee et al. have achieved a detection accuracy of 100% in a study focusing on moderate HCC (mean diameter of 3.1 cm) and small HCC (mean diameter of 1.04 cm), in comparison to traditional radiological diagnosis [88].

The previous paragraphs highlight the potential of AI to enhance the accuracy and efficiency of HCC screening and detection, especially with data integration across various modalities. Most studies have focused on the diagnosis, treatment response and prognosis of HCC, while there is a shortage of studies utilizing AI for HCC screening. A majority of the studies that have been conducted for the diagnosis of HCC are retrospective studies, which can affect the diagnostic accuracy of the studies. There is a need for robust, large prospective studies to validate the findings of these studies so that the technologies are brought into the mainstream and positively impact patient management [83]. The ideal AI-based protocol for HCC screening should integrate clinical data with laboratory and radiological findings to maximize accuracy.

Cost-effectiveness is considered critical for it to be acceptable. Therefore, screening is recommended in a specific population with chronic liver disease at the highest risk for HCC. The recommendations from the EASL and AASLD differ in patients with Hepatitis C with F3 fibrosis and cirrhosis. The EASL defines F3 fibrosis based on the fibroscan (10–13 kPa) or ARFI (VTQ) of 1.6–2.17 m/s, while the AASLD does not provide specific values for the F3 fibrosis. Further, the EASL recommends every 6 months US for patients with non-cirrhotic F3 fibrosis, while the AASLD does not recommend such ultrasounds [89]. Although HCC can occur in patients with less severe chronic liver disease of different ideologies, the different societies do not advocate HCC screening in these populations as the incidence of HCC is much lesser when compared to the population who satisfy the criteria for HCC screening. In the future, AI algorithms could assist in the development of cost-effective HCC screening programs in this subset of the population which could be incorporated in current screening strategies.

Although screening modalities with intervals have been well studied in HCC patients, individuals with non-cirrhotic HCC (NCHCC) are often diagnosed late in the disease process, likely due to challenges in recognizing and screening. The prevalence of NCHCC could be as high as 20% among all patients with HCC. The risk factors for the development of the NCHCC include viral factors (hepatitis B and hepatitis C without cirrhosis) and non-viral factors (obesity, diabetes, toxin exposure [alcohol, aflatoxins, anabolic steroids, genotoxins], hereditary hemochromatosis, glycogen storage disorders, acute hepatic porphyria’s, genetic disorders). It is important to recognize the imaging features of NCHCC vary compared to CHCC patients. The patients with NCHCC are noted to have on CT scan (large solitary mass with satellite nodules, extrahepatic extension with increased risk of metastasis and low risk of vascular invasion) and MRI (T2 hyperintense and T1 unenhanced lesion and low apparent diffusion coefficient on diffuse weighted imaging) compared to CHCC [90]. Patients with hepatitis C who have advanced fibrosis (such as F3 or beyond) are at increased risk of NCHCC and hence should qualify for screening. Further, achieving sustained virological response (SVR) after anti-viral agents reduces the risk (up to 70%) but does not eliminate it completely [89]. Although anti-viral treatment reduces the viral load, the fibrosis takes a significantly longer time to reverse. Further, ongoing genetic, epigenetic, and micronodule-related changes still predispose to hepatocyte injury, leading to a neoplastic process in the future. Hence, the patients with F3 or beyond and with other risk factors should be carefully assessed. The EASL guidelines recommend that HCV patients, after achieving SVR, may be considered for surveillance based on the risk assessment [74]. However, the AASLD does not recommend surveillance in non-cirrhotic HCV patients [51]. This remains a gap in the screening strategy for these patients, likely due to difficulty of accurately determining the F2 vs. F3 fibrosis and understaging fibrosis with non-invasive markers. Hence, If CT or MRI is equivocal in nature, the use of histological assessment might be required. This could not only assess the degree of fibrosis but also identify potentially early features of HCC. The American gastroenterology association (AGA) recommends surveillance of metabolic dysfunction-associated steatotic liver disease (MASLD) patients with non-invasive markers of fibrosis or cirrhosis to assess the need for further imaging [91]. This could increase the detection of early cases of NCHCC and hence could overall increase the rate of early identification of these patients. Nevertheless, the use of non-invasive markers combined with high-quality cross-sectional imaging should be incorporated in patients with risk factors for HCC, even without cirrhosis.

## 12. Confirmation of the Diagnosis of HCC

Imaging is integral to the diagnosis of HCC in the patient population with chronic liver disease who meet the criteria for HCC screening, i.e., established cirrhosis and a subset of the population with chronic HBV infection. When the typical imaging features of HCC are seen in these patients, imaging alone is enough to confirm the diagnosis [43]. Multiphasic CT, contrast-enhanced MRI, and contrast-enhanced ultrasounds are imaging modalities available for the diagnosis of HCC.

Multiphasic CT: A multiphasic CT of the abdomen for the evaluation of HCC includes four phases: the non-contrast phase, the late arterial phase (25–30 s after contrast injection), the portal venous phase (60–80 s after contrast injection), and the equilibrium phase (2–5 min after contrast injection). The slice thickness should be less than 5 mm, and the rate of injection should be greater than 3 mL/s, followed by a saline flush. Optimal timing, especially of the late arterial phase, is critical for the diagnosis of HCC as arterial phase hyper-enhancement is the most important diagnostic feature of an HCC. Factors that can influence the contrast timing include variations in cardiac output or IV catheter-related issues. The bolus-tracking technique is the most frequently used technique to optimize the acquisition timing [92,93].

MRI: MRI, like CT, predominantly relies on multiphase pre- and post-contrast imaging for the diagnosis of HCC, which is obtained as T1 pre-contrast, late arterial phase, portal venous phase, and delayed phase images. The timing of the multiple post-contrast acquisitions is similar to that of the multiphasic CT study. Akin to the CT acquisition, the timing of the late arterial phase is critical, and bolus tracking is recommended for precise contrast timing [94]. In addition to the pre- and post-contrast T1-weighted images, in-phase and out-of-phase T1-weighted images, as well as T2-weighted images, are obtained. Diffusion-weighted images are optional. The MRI sequences performed in addition to the pre and post-contrast T1-weighted images are helpful in further lesion characterization. MRI is advantageous compared to CT as it avoids radiation. The limitations include longer acquisition times, dependence on patient breath holding, and dielectric artifacts that degrade the images if there is a moderate to large volume of ascites [95].

**Figure 3:**
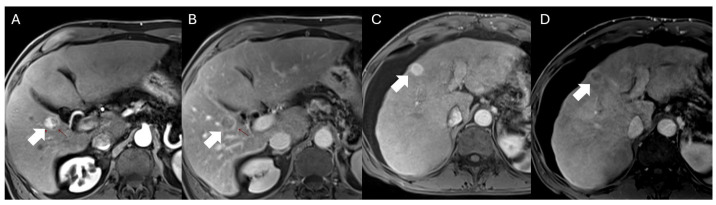
Images (**A**,**B**) axial post-contrast T1 arterial and delayed post-contrast images demonstrate arterial enhancing lesion demonstrating non-peripheral washout and delayed capsular enhancement, LR-5 observation, consistent with hepatocellular carcinoma (arrows). Images (**C**,**D**) axial post-contrast T1 arterial and delayed post-contrast images in a different patient demonstrate arterial enhancing lesion demonstrating non-peripheral washout and delayed capsular enhancement, LR-5 observation, consistent with hepatocellular carcinoma (arrows).

The Liver Imaging Reporting and Data System (LI-RADS) has been proposed by the American College of Radiology for the characterization of hepatic observations on multiphasic CT or MRI studies. This system applies only to adults with cirrhosis and chronic HBV even without cirrhosis [27,96]. Multiphasic CT and MRI examinations include images acquired before and following intravenous contrast administration, as detailed above [97]. Arterial phase hyperenhancement is the hallmark of HCC. The LI-RADS scoring system utilizes this feature along with the size of the observation and other significant features demonstrated by HCC, including non-peripheral washout, delayed capsular hyperenhancement, and threshold growth (Figure 3). The observations range from LR-1 to LR-5. Based on the imaging characteristics, observations can be categorized as LR-1: definitely benign, LR-2: probably benign, LR-3: indeterminate, LR-M: probably or definitely malignant but not specific for HCC, LR-4: probably HCC and LR-5: definitely HCC. An LR-5 lesion diagnosed on CT or MRI examinations has a 95–99% chance of being an HCC [43,96].

The introduction of contrast agents marked a significant turning point in the ultrasonographic diagnosis of HCC [55]. Contrast-enhanced ultrasounds utilize gas microbubbles (stable perfluorocarbon or sulfur hexafluoride) enclosed in an albumin or phospholipid shell. It is a noninvasive modality that allows for real-time assessment of the hepatic lesion’s perfusion. Contrast-enhanced ultrasounds (CEUS) typically employs SonoVue^®^ (Sulfur hexafluoride), a contrast agent not absorbed by Kupffer cells, which facilitates the observation of arterial, portal-venous, and late arterial phases lasting up to 6 min [98]. The characteristic feature of hepatocellular carcinoma (HCC) on CEUS using SonoVue^®^ is a uniform and intense arterial phase hyper-enhancement (APHE) with mild washout beginning more than 60 s post-injection [99]. According to the guidelines from the European Federation of Societies for Ultrasound in Medicine and Biology (EFSUMB), CEUS can be utilized for cases with inconclusive CT or MRI results, in patients who are not candidates for biopsy, and for tracking changes in enhancement in nodules under surveillance [98]. The advantages of CEUS include real-time scanning that optimizes the detection of APHE with high sensitivity, the ability to precisely time the arrival of microbubbles precisely (indicating arterialization) or washout—both potential indicators of malignancy, suitability for free-breathing, which enhances patient comfort, and the use of a non-nephrotoxic contrast agent. However, disadvantages involve a focused field-of-view that may limit the staging of the entire liver, operator dependency, and limitations due to patient body habitus [55,100].

As per the AASLD guidelines, when typical imaging features are present in at-risk patients, a diagnosis of HCC can be established without histopathological confirmation. MRI and CT are equally recommended by the Society for the confirmation of diagnosis, although MRI has a higher sensitivity with a similar specificity when compared to CT. As per the Society’s recommendations, CEUS could be used as a second line and imaging modality in case MRI and CT are not able to provide a definitive diagnosis, cannot be performed, or are unavailable, especially in a setting where it is not possible to perform a biopsy. The Society also recommends the usage of the LI-RADS algorithm for the characterization of hepatic lesions in at-risk patients. In patients with liver nodules suspicious of HCC without cirrhosis or chronic HBV infection or when imaging is inconclusive, a liver biopsy is needed to obtain a pathological diagnosis. If the biopsy is negative, it does not rule out the possibility of an HCC, and a second biopsy needs to be performed if the findings are negative or equivocal. LI-RADS criteria consider tumor size as a criterion for the diagnosis of HCC due to decreased accuracy for imaging diagnosis of lesions measuring less than 2 cm in size [43].

EASL agrees that HCC can be diagnosed in patients with cirrhosis based on imaging when the characteristic features are present and that the accuracy of imaging is decreased in tumors measuring less than 2 cm. EASL also recommends CEUS as a second-line imaging modality if the CT and MRI are inconclusive, cannot be performed, or are contraindicated [74].

## 13. Limitations

Screening for HCC is primarily based on imaging. A large number of patients with chronic liver disease are asymptomatic and are therefore not subject to screening, which prevents early identification. Further, chronic liver disease, without the development of cirrhosis, constitutes up to 20% of the HCC patient population, which could be potentially missed. Challenges such as suboptimal visualization of the liver parenchyma limit the capability of the ultrasound as the screening modality. Further research and policies are needed so that screening can be performed in the patient population with chronic liver disease who do not have cirrhosis without causing an economic burden to the patients and the community. The use of combination testing, such as biomarkers with imaging, could reduce the risk of missing lesions, especially in patients with early-stage cirrhosis. Additionally, AI modeling could add capabilities to identify these patients by analyzing and integrating large data sets. However, these applications will need validation for a wide variety of patient populations, which will remain to be studied in the future.

## 14. Conclusions

HCC is among the most common cancers and among the leading causes of cancer-related mortality. It typically occurs in the setting of chronic liver disease. The early diagnosis of HCC helps in prompt management and preventing morbidity and mortality. In patients with cirrhosis and select patients with chronic HBV infection, using ultrasounds on a six-month basis and serum AFP levels is the most accepted screening tool for early diagnosis. The role of ultrasounds is to detect focal abnormality, which is further characterized by cross-sectional imaging. CT or MRI can be used as a screening tool if limitations are encountered during ultrasounds. Multiphasic CT or MRI are also useful in confirming the diagnosis of HCC.
